# Dielectric and Viscoelastic Behavior of Polyvinyl Butyral Films

**DOI:** 10.3390/polym15244725

**Published:** 2023-12-16

**Authors:** Jesús G. Puente-Córdova, Flor Y. Rentería-Baltiérrez, Beatriz López-Walle, Juan A. Aguilar-Garib

**Affiliations:** 1 Facultad de Ingeniería Mecánica y Eléctrica, Universidad Autónoma de Nuevo León, Av. Universidad s/n, Cd. Universitaria, San Nicolás de los Garza 66455, Mexico; 2 Facultad de Ciencias Químicas, Universidad Autónoma de Nuevo León, Av. Universidad s/n, Cd. Universitaria, San Nicolás de los Garza 66455, Mexico

**Keywords:** dielectric properties, polyvinyl butyral, electric modulus, fractional calculus, relaxation phenomena

## Abstract

Dielectric and thermal properties of polyvinyl butyral (PVB) were studied in this work, using dynamic electrical analysis (DEA) at frequencies from 100 Hz to 1 MHz and temperatures from 293 K to 473 K. Two electrical relaxation processes were investigated: glass transition and interfacial polarization. Above the glass transition temperature (~343 K), interfacial polarization dominates conductive behavior in polyvinyl butyral. The framework of the complex electric modulus was used to obtain information about interfacial polarization. The viscoelastic behavior was analyzed through dynamic mechanical analysis (DMA), where only the mechanical manifestation of the glass transition is observed. The experimental results from dielectric measurements were analyzed with fractional calculus, using a fractional Debye model with one cap-resistor. We were successful in applying the complex electric modulus because we had a good correlation between data and theoretical predictions. The fractional order derivative is an indicator of the energy dissipated in terms of molecular mobility, and the calculated values close to 1 suggest a conductive behavior at temperatures above the glass transition temperature of PVB.

## 1. Introduction

Electrical conductivity, permittivity, and dielectric strength are among the most important properties of materials for the electrical industry. In the case of polymers, they are widely applied as electrical insulators or dielectrics, and seldom as semiconductors. However, there are new synthesized polymers with a specific molecular structure that can be converted into electrically conductive materials [1,2,3]. Some structures are far from thermodynamic equilibrium, thus physical aging affects the electrical properties of polymers [4,5]. Physical aging is a phenomenon associated with structural recovery or relaxation of the glassy state towards the metastable equilibrium of the amorphous state, presenting important changes in physical properties as a function of temperature and time. The accumulation of electrical charges on the surface (electrodes) or bulk (traps) contributes to the physical aging of polymers under electrical fields. This generates a scenario that produces space charge relaxation, also known as interfacial polarization or Maxwell–Wagner–Sillars polarization [6,7]. Some experiments have been performed on space charge relaxation above the glass transition temperature (Tg). Among them, there are some works that use dynamic electrical analysis (DEA) to study the space charge relaxation or interfacial polarization in polymers [8,9,10,11]. It is established that above the Tg and at low frequencies (typically 10^−3^–10^2^ Hz), extrinsic and intrinsic carriers contribute to the generation of interfacial polarization. Since these observations are not easy to obtain in the spectrum of complex relative permittivity $\varepsilon_{r}^{*}$ using DEA, the use of the complex electric modulus $M^{*}$ has been proposed as an alternative [12,13].

The concept of electric modulus was first proposed by McCrum et al. [13,14] and intensively used for the investigation of the electrical relaxation phenomena of organic dielectric materials [8,9,12,13,14]. This is analogous to the complex elastic modulus used in linear viscoelasticity. $M^{*}$ represents the electric field relaxation in the dielectric material when the electric displacement remains constant. Richert & Wagner [15] present experimental results about $M^{*}$ considering it as a true dielectric relaxation, while $\varepsilon_{r}^{*}$ refers to the dielectric retardation process. $M^{*}$ is calculated as the inverse of $\varepsilon_{r}^{*}$, Equation (1):

(1) $$M^{*}=M^{\prime }+M^{\prime \prime }=\frac{1}{\varepsilon_{r}^{*}}=\frac{1}{\varepsilon_{r}^{\prime }-i\varepsilon_{r}^{\prime \prime }}=\frac{\varepsilon_{r}^{\prime }}{{\left(\varepsilon_{r}^{\prime }\right)}^{2}+{\left(\varepsilon_{r}^{\prime \prime }\right)}^{2}}+i\frac{\varepsilon_{r}^{\prime \prime }}{{\left(\varepsilon_{r}^{\prime }\right)}^{2}+{\left(\varepsilon_{r}^{\prime \prime }\right)}^{2}}$$

where $M^{\prime }$ is the real part and $M^{\prime \prime }$ the imaginary part of the complex electric modulus, and $\varepsilon_{r}^{\prime }$ is the real part and $\varepsilon_{r}^{\prime \prime }$ the imaginary part of the complex relative permittivity.

Considering this knowledge, in this study we investigate the thermal and dielectric response of polyvinyl butyral (PVB). We are interested in this polymer due to its potential applications, such as for hybrid magnetic materials and nanocomposites, another topic of our research group [16,17,18]. PVB is a polyacetal considered as an amorphous random copolymer with viscoelastic properties and optical clarity, suitable for use in the manufacture of safety glass laminates, especially in automotive and architectural glass [19,20]. PVB is obtained by modifying polyvinyl alcohol by condensation with butyraldehyde in an acid media. The result of this chemical modification produces polymeric chains whose structure is formed by three structural units along the PVB chains: butyral, alcohol, and acetate units [21,22]. The butyral unit is hydrophobic, and the alcohol unit is considered hydrophilic, giving in the adhesion property to be used as coating on wood, glass, or metals.

Regarding studies of electrical properties of PVB, most were carried out below Tg (~343 K). For instance, Jain et al. carried out the study of the electrical properties of PVB using the thermally stimulated discharge currents (TSDC) technique [23]. They observed two relaxation phenomena, the first one at a temperature of 347 K and the second one at 423 K. They attributed the first phenomenon to the disorientation of electric dipoles, and the second phenomenon to the release of electric charges trapped in the PVB. Some authors [24,25,26] worked on the dielectric properties of PVB, around the glass transition temperature, by measuring $\varepsilon_{r}^{*}$. As a general conclusion of these studies, the main relaxation consists of a thermally activated process, where electric dipoles of various chemical groups of the polymer chains participate. Carine et al. [27] studied the secondary and principal relaxations of PVB using dielectric spectroscopy, for $\varepsilon_{r}^{\prime }$ and $\varepsilon_{r}^{\prime \prime }$. It was observed that when the temperature increased from 120 to 250 K, $\varepsilon_{r}^{\prime \prime }$ increases and passes through the secondary β-relaxation peak at about 190 K. Above this region, $\varepsilon_{r}^{\prime \prime }$ shows the α-relaxation peak at approximately 340 K, which is associated with the glass transition phenomenon.

The objective of this work is to evaluate the thermal, viscoelastic, and dielectric properties and the interfacial polarization of PVB films at temperatures above Tg. The novelty of this study lies in applying the fractional calculus approach under the theoretical framework of the electric modulus. The main feature of PVB is that it is a good alternative to perform functions as organic insulator or dielectric material in technological devices such as solar cells, electronic components, and supercapacitors, among others.

## 2. Materials and Methods

### 2.1. Preparation of Polyvinyl Butyral Films

Polymer samples were manufactured as thin films from solutions of polyvinyl butyral (PVB-BM, Mw = 53,000 g/mol, Sekisui, Osaka, Japan) in tetrahydrofuran (THF, 99%, Sigma-Aldrich, St. Louis, MI, USA) as a solvent. A concentration of 10% by weight was used, which ensures an appropriate viscosity for the manufacture of the films. To guarantee the dissolution process, the PVB-THF mixture was stirred at 700 rpm for 1 h at 313 K. The solutions obtained were tape-casted on a PTFE surface, after which the solvent was removed by natural convection at room temperature for 24 h. The thickness of the obtained films was ~20 μm.

### 2.2. Thermal, Viscoelastic, and Dielectric Characterization

To determine the thermal response of the PVB, thermogravimetric analysis (TGA) was carried out in an air atmosphere at a heating rate of 10 K/min in a temperature range between 295 and 873 K. The viscoelastic response of the PVB was analyzed by dynamic mechanical analysis (DMA) using a Perkin Elmer DMA8000 (Waltham, MA, USA), in tension mode, for three frequency values (0.1 Hz, 1 Hz and 10 Hz). The sample was subjected to a controlled strain with an amplitude of 0.01 mm, in a temperature range from 300 to 443 K, using a 2 K/min heating rate. By this isochronal condition, the storage modulus $E^{\prime }$ and loss modulus $E^{\prime \prime }$ were registered. Tangent delta was calculated as $\tan\delta_{m}=E^{\prime \prime }/E^{\prime }$. The dimensions of the analyzed films were 20 mm in length, 7 mm in width, and a thickness of 20 μm.

Dynamic electrical analysis (DEA) measurements were taken in a frequency interval from 100 Hz to 1 MHz using an Agilent E4980A electrometer (Santa Clara, CA, USA). The PVB sample was placed between two electrodes of copper. The applied voltage that defines the electric field oscillates between −1 and 1 V, and the measurements were carried out at different temperatures, from 293 to 473 K in steps of 10 K. To corroborate that space charge relaxation manifests at high temperatures, electrical current measurements were fulfilled using the technique identified as thermally stimulated discharge current (TSDC). For this purpose, a polarization voltage Vp = 500 V in direct current was applied, for a polarization temperature Tp = 363 K in a time interval of 1200 s. The sample was cooled rapidly (~20 K/min). Then it was short-circuited between the electrodes, and the recording of the electric current was started from 303 K. A heating rate of 5 K/min was used, and a Keithley 6517B electrometer (Beaverton, OR, USA) with a precision of 10^−14^ A was used for the TSDC measurements.

### 2.3. The Fractional Debye Model and the Cap-Resistor

**Figure 1:**
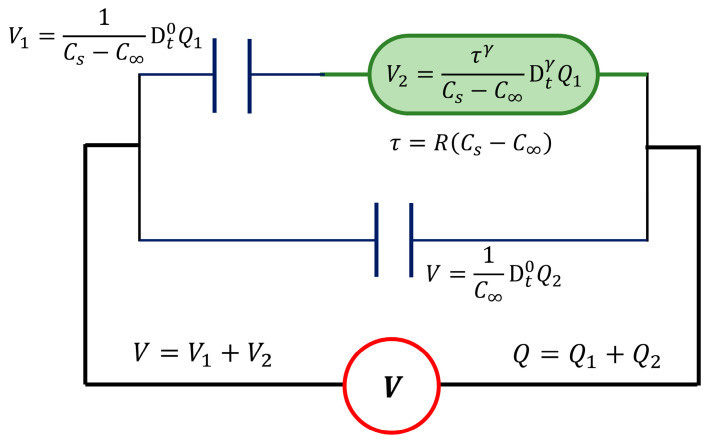
The fractional Debye model with one cap-resistor.

Nowadays, fractional calculus is a branch of mathematics that deals with the derivatives and integrals of arbitrary order [28] and offers diverse applications in science and engineering. In the field of rheology, fractional operators are used to model the mechanical behavior of polymeric systems, where there is a partial storage or dissipation of energy. Regarding the study of the dielectric behavior of materials, the empirical Cole-Cole, Cole–Davidson, and Havriliak–Negami equations were used for the interpretation and analysis of experimental data. However, full precision has not yet been achieved. In previous studies it has been shown [29,30,31] that by using fractional calculus, the dielectric behavior of organic materials can be described with a higher degree of accuracy than by using integer order calculus. In this sense, we use an electrical element, the “cap-resistor” [32,33], which is analogous to the spring-pot or Scott Blair element used in linear viscoelasticity. The cap-resistor, Equation (2), intimately combines the electrical response of a capacitor (energy storage) with the response of a resistor (energy dissipation) by a differential operator of fractional order γ:

(2) $$V=\frac{\tau^{\gamma }}{C}D_{t}^{\gamma }Q=\frac{{\left(\mathrm{RC}\right)}^{\gamma }}{C}D_{t}^{\gamma }Q$$

where V is the voltage, Q is the electric charge, C is the capacitance, and R is the resistance. $D_{t}^{\gamma }Q$ is the fractional derivative operator of the Q, with 0≤γ≤1. The electric relaxation time τ=RC can be associated with the time required by electric charge carriers in movement for a complete reorganization to a new equilibrium state. When γ=0 in Equation (2), the capacitance or dielectric response is obtained; otherwise, when γ=1, the electrical resistance response is obtained. The fractional derivative operator, used in Equation (2), can be expressed by the Riemann-Liouville definition, Equation (3):

(3) $$D_{t}^{\gamma }Q=\frac{1}{\Gamma \left(1-\gamma \right)}\frac{d}{\mathrm{dt}}\int_{0}^{t}\frac{Q\left(\xi \right)}{{\left(t-\xi \right)}^{\gamma }}d\xi$$

with 0<γ<1, and Γ(·) is the gamma function. The fractional order derivative can be related to the partial energy dissipated by the system; several studies have shown that fractional orders are related to the molecular mobility of organic dielectric materials [17,29]. Therefore, the equivalent electrical circuit of the classical Debye model was modified by replacing the resistor with one cap-resistor, γ. Figure 1 shows the components that describe this fractional model. The cap-resistor, γ, characterizes times (τ) associated with dielectric response in the region around dielectric relaxation, and the two capacitor elements represent the storage dielectric response. $C_{s}$ is the capacitance at low frequencies or high temperatures, and $C_{\infty }$ is the capacitance at high frequencies or low temperatures.

Then, from the constitutive equations of the capacitors and the cap-resistor, the fractional differential equation can be obtained, Equation (4), with 0<γ≤1:

(4) $$V=\frac{Q-C_{\infty }V}{C_{s}-C_{\infty }}+\frac{\tau^{\gamma }}{C_{s}-C_{\infty }}D_{t}^{\gamma }\left[Q-C_{\infty }V\right]$$

From Equation (4), the complex dielectric permittivity $\varepsilon_{r}^{*}$ is calculated as a function of the angular frequency ω, under isothermal conditions, see Equation (5). The Fourier transform of the fractional derivative $D_{t}^{\gamma }$ is obtained as the product of ${\left(i\omega \right)}^{\gamma }$ by the Fourier transform of the function [29,31].

(5) $$\varepsilon_{r}^{*}=\frac{\varepsilon_{\mathrm{rs}}+\varepsilon_{r\infty }{\left(i\omega \tau \right)}^{\gamma }}{1+{\left(i\omega \tau \right)}^{\gamma }}$$

where $\varepsilon_{\mathrm{rs}}$ is the permittivity at low frequencies or high temperatures and $\varepsilon_{r\infty }$ is the permittivity at high frequencies or low temperatures. It is interesting that Equation (5) also corresponds to the empirical Cole-Cole function used to fit the dielectric data. Furthermore, it is possible to obtain a mathematical expression for the complex electric modulus $M^{*}$ from Equation (5), as pointed out in Equation (1). The result is presented in Equation (6).

(6) $$M^{*}=M^{\prime }+{\mathrm{iM}}^{\prime \prime }=\frac{1}{\varepsilon_{r}^{*}}={\;M}_{\infty }M_{s}\frac{1+{\left(i\omega \tau \right)}^{\gamma }}{M_{\infty }+{\;M}_{s}{\left(i\omega \tau \right)}^{\gamma }}$$

Here, $M_{\infty }=1/\varepsilon_{r\infty }$ is the electric modulus at high frequencies and $M_{s}=1/\varepsilon_{\mathrm{rs}}$ is the electric modulus at low frequencies. Equations (7) and (8) show the mathematical expressions for $M^{\prime }$ and $M^{\prime \prime }$, respectively, which were obtained from Equation (6).

(7) $$M^{\prime }=M_{\infty }M_{s}\frac{M_{\infty }+\left(M_{s}+{\;M}_{\infty }\right)\left[\cos\left(\gamma \frac{\pi }{2}\right){\left(\omega \tau \right)}^{\gamma }\right]+M_{s}{\left(\omega \tau \right)}^{2\gamma }}{M_{\infty }^{2}+2M_{s}M_{\infty }\left[\cos\left(\gamma \frac{\pi }{2}\right){\left(\omega \tau \right)}^{\gamma }\right]+M_{s}^{2}{\left(\omega \tau \right)}^{2\gamma }}$$

(8) $$M^{\prime \prime }=M_{\infty }M_{s}\frac{\left(M_{\infty }-M_{s}\right)\left[\sin\left(\gamma \frac{\pi }{2}\right){\left(\omega \tau \right)}^{\gamma }\right]}{M_{\infty }^{2}+2M_{s}M_{\infty }\left[\cos\left(\gamma \frac{\pi }{2}\right){\left(\omega \tau \right)}^{\gamma }\right]+M_{s}^{2}{\left(\omega \tau \right)}^{2\gamma }}$$

It is important to highlight that the fractional order derivative γ can only take values between 0 and 1. When γ=1, we recover the classical equations for the Debye model (single relaxation process). Equations (7) and (8) correspond to a symmetric response, which is typical of interfacial polarization or space charge relaxation [11,12]. From imaginary modulus $M^{\prime \prime }$, Equation (8), it is possible to establish a relationship from the maximum peak to estimate the relaxation time $\tau_{M}=\tau {\left(M_{s}/M_{\infty }\right)}^{1/\gamma }$, where τ is defined as mentioned above. The loss tangent is calculated as $\tan\delta_{e}=M^{\prime \prime }/M^{\prime }$.

## 3. Results and Discussion

### 3.1. Thermal and Viscoelastic Analysis

**Figure 2:**
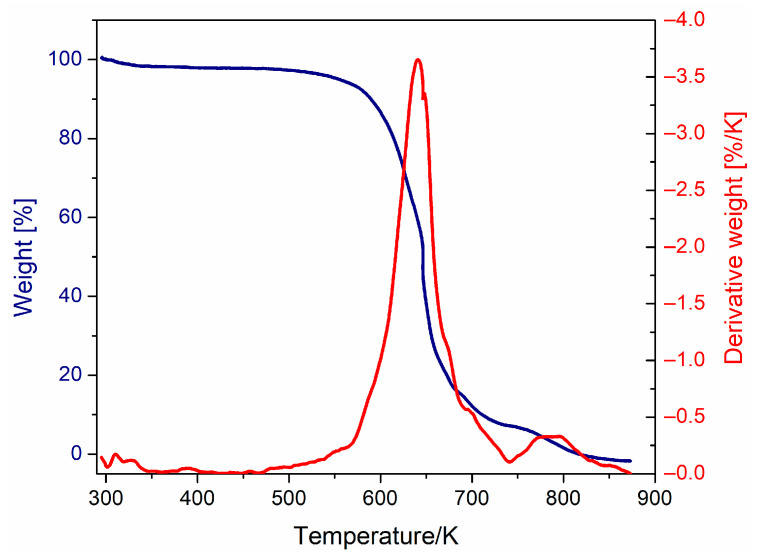
Thermogravimetric curves of PVB.

Thermal stability and degradation of PVB due to the heating effect were determined by means of TGA. Figure 2 shows the thermogravimetric curve obtained. The degradation temperature is identified at 641 K, whose decomposition products correspond mainly to butyraldehyde and acetic acid, due to the degradation of butyral and acetate units of polymer chains, respectively. At a temperature above 741 K, the sample degrades completely [34,35]. The presence of water was not detected, since no significant decrease in weight loss at the boiling temperature was observed. These results are important because they impact the electrical response and must be considered in the dielectric measurements above the Tg of PVB.

**Figure 3:**
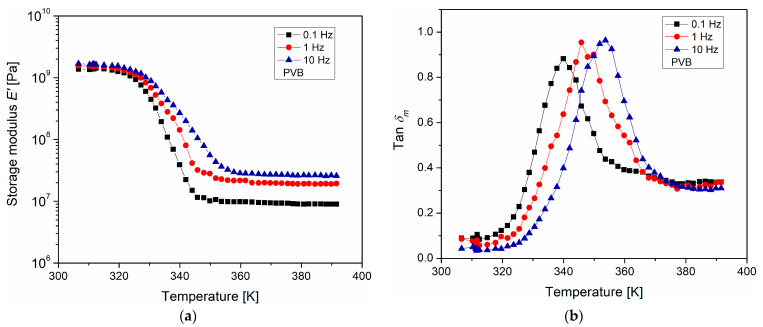
(**a**) storage modulus and (**b**) $\tan\delta_{m}$, as a function of temperature and frequency, for PVB.

The mechanical manifestation of the relaxation phenomena of the PVB was analyzed using DMA, in a temperature range from 303 to 393 K. Figure 3a presents the graph of the storage modulus $E^{\prime }$ as a function of temperature. A decrease in $E^{\prime }$ was observed when the temperature increased; this is associated with the α-relaxation, which corresponds to the mechanical manifestation of the glass transition, and is correlated with the cooperative mobility of the chain segments. This process corresponds with a notable increase in molecular mobility when the temperature increases and with long-range molecular movements at temperatures around the glass transition temperature Tg. At temperatures above 353 K, a region in which $E^{\prime }$ remains constant, but sensitive to frequency, was identified. This region was found to be elastomeric, occurs at temperatures above Tg, and is a function of the number of physical crosslinks generated between the polymer chains and chemical groups. At higher temperatures, the polymer begins to flow. In Figure 3b, $\tan\delta_{m}$ is shown as a function of temperature for each of the analyzed frequencies. In these plots, $\tan\delta_{m}$ is the ratio between the energy dissipated and the energy stored by the PVB. The maximum or peak of $\tan\delta_{m}$ corresponds to the maximum energy dissipation of the main PVB relaxation process. The temperature at which these peaks occur is estimated as the Tg; and, when the frequency increases, the $\tan\delta_{m}$ peaks shift toward higher temperatures. The estimated Tg values were 340 K, 346 K, and 353 K for the frequencies of 0.1 Hz, 1 Hz, and 10 Hz, respectively. Additionally, a differential scanning calorimetry (DSC) measurement was carried out to calculate the Tg of the PVB, whose value was 333 K. These values are consistent with those reported by Calucci et al. [36] and by Kirchberg et al. [37]. In DMA, the stimulus comes from a surface force, which generates significant mobility in the polymer chains of PVB. Through DEA, it is possible to analyze the molecular mobility in a more selective way, since a body force (electric field) is applied and acts on the electric charge carriers, electrons, ions, and electric dipoles.

### 3.2. Dynamic Dielectric Analysis DEA

**Figure 4:**
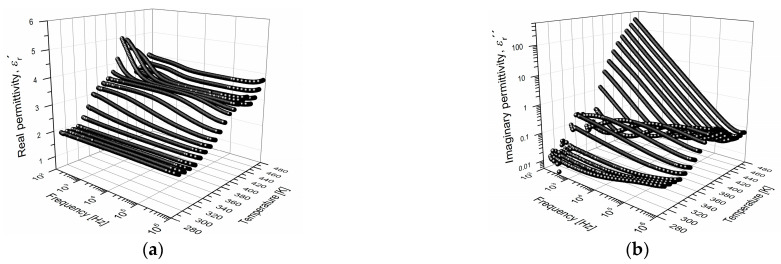
(**a**) real permittivity $\varepsilon_{r}^{\prime }$ and (**b**) imaginary permittivity $\varepsilon_{r}^{\prime \prime }$, as a function of frequency and temperature, for PVB.

The measurements of real $\varepsilon_{r}^{\prime }$ and imaginary relative permittivity $\varepsilon_{r}^{\prime \prime }$ were obtained as a function of frequency in an interval from 100 Hz to 1 MHz, for different constant temperatures. Figure 4 shows the isothermal curves obtained. Regarding the isothermal curves of $\varepsilon_{r}^{\prime }$ (Figure 4a), at temperatures above 353 K, as the frequency increases, the magnitude of $\varepsilon_{r}^{\prime }$ decreases. This decrement is more pronounced as the temperature increases. Among the electrical phenomena that must define the shape of these curves, there is, in addition to the α-relaxation (electrical manifestation of the glass transition), the interfacial polarization and the conductivity process. These phenomena are strongly influenced by the polymer structure and morphology [5,31]. Conductive processes, ionic conduction, and interfacial polarization must occur at temperatures above the Tg [8,10,12]. However, it is a complex process to separately identify the presence of any electrical relaxation phenomena.

Components of the complex permittivity, $\varepsilon_{r}^{\prime }$ and $\varepsilon_{r}^{\prime \prime }$, depend on the electric dipoles of the PVB. The value of $\varepsilon_{r}^{\prime }$ depends directly on the number of them that are oriented according to the intensity and direction of the applied electric field. The dipoles that present significant mobility are the hydroxyl groups (~1.6 D), since acetals have greater mass and volume, presenting restricted mobility [22,38]. Instead, as the frequency of the applied electric field increases, there is not enough time to be aligned by the field so that the number of oriented dipoles decreases, which is reflected in the magnitude of $\varepsilon_{r}^{\prime }$. At low frequencies and above the Tg, an increase in the value of $\varepsilon_{r}^{\prime }$ was observed, which represents a greater electrical charge storage capacity. In the curves of $\varepsilon_{r}^{\prime \prime }$ (Figure 4b), as the temperature increases, a broad relaxation peak was observed. This peak shifts towards high frequencies as the temperature increases, corroborating the presence of electrical phenomena that are constituent by thermally activated movements. The relaxation peaks are associated with a distribution of relaxation times of the electric dipoles of the PVB and correspond mainly to the electrical manifestation of the glass transition. In Figure 4b, when the temperature increases above the Tg, a behavior of type $\varepsilon_{r}^{\prime \prime }\propto 1/f$ at low frequencies is observed, resulting [27,36] in ionic conductivity due to the rubbery state and the activation of the micro-Brownian motion of the polymer segments. This observed behavior is consistent with various reports for polymers like PMMA, PS, PET, and PEEK [8,10,11,12]. However, in this temperature region, interfacial polarization can also manifest itself and thus overlap with the ionic conductivity. Therefore, the data are approached using the theoretical framework of the electric modulus $M^{*}$.

### 3.3. Electric Modulus Approach

**Figure 5:**
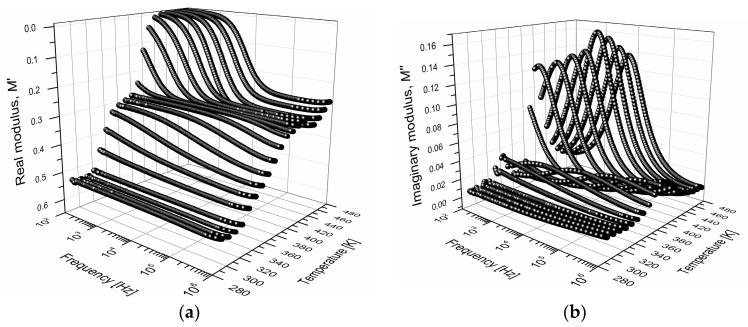
(**a**) real modulus $M^{\prime }$ and (**b**) imaginary modulus $M^{\prime \prime }$, as a function of frequency and temperature, for PVB. The axis of $M^{\prime }$ has been inverted for better visualization.

Using the Equation (1), $M^{\prime }$ and $M^{\prime \prime }$ were calculated from the Figure 4a,b. In Figure 5a the isothermal curves for $M^{\prime }$ are presented, and in Figure 5b those obtained for $M^{\prime \prime }\;are\;presented$. These curves exhibit shapes close to the typical curves of electrical relaxation phenomena in polymeric materials [13,16]. In Figure 5a, $M^{\prime }$ increases as the frequency increases and becomes more pronounced as the temperature increases, which is the opposite to what was observed for $\varepsilon_{r}^{\prime }$. The increment of $M^{\prime }$ with a frequency at temperatures above Tg corresponds to a maximum or peak in the curves of $M^{\prime \prime }$.

In the $M^{\prime \prime }$ curves (Figure 5b), two types of relaxation are distinguished. Some peaks located in a temperature interval of 293–373 K are identified and correspond to the electrical manifestation of the glass transition of the PVB (α-relaxation). The peaks identified in the temperature interval 393–473 K correspond to the interfacial polarization, which is consistent with the results obtained by Jain et al. [23]. The interfacial polarization peak is only detectable in electrical measurements and not from DMA, DSC, or TGA. The interfacial polarization phenomenon occurs through the accumulation of electrical charge in regions of the electrode-polymer interface or within the polymer bulk [8,39,40]. This process is due to the complex structure of the PVB, an amorphous copolymer, which presents traps with different energy levels (shallow and deep traps). For PMMA and PEI it was reported that the physical model of Coelho and the macrodipole concept are utilized to explain the space charge relaxation or interfacial polarization that occurs at temperatures above the Tg [8,12]. In Figure 5b, the relaxation peaks shift toward high frequencies as the temperature increases [8,9,41].

**Figure 6:**
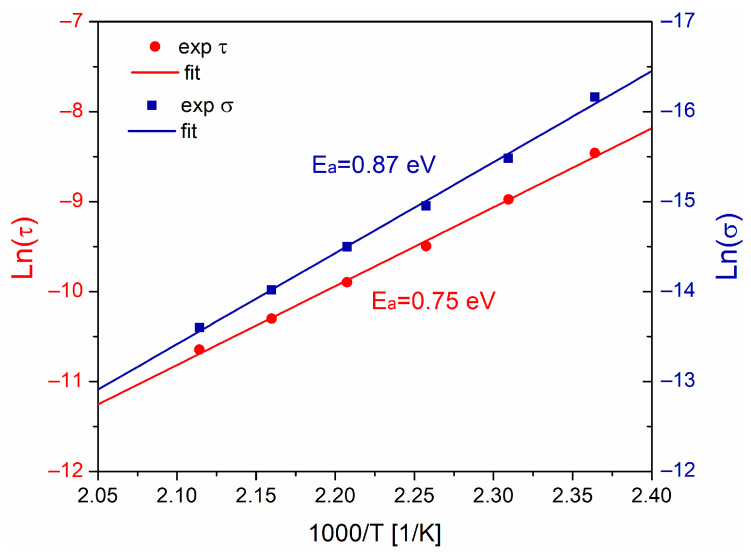
Arrhenius plot of relaxation time and conductivity for PVB.

From Figure 5b, the activation energy ($E_{a}$) for interfacial polarization was calculated, using the relation $\tau =\exp(-E_{a}/\mathrm{kT})$, by plotting ln(τ) against 1000/T (Figure 6). The value obtained for $E_{a}=$ 0.75 eV, consistent with others results reported for PMMA, epoxy resin, EPDM, among others [12,14,42]. This is higher compared to the activation energy calculated from α-relaxation ($E_{a}=$ 0.45 eV). The contribution of conductivity σ at low frequencies was estimated from Figure 4b using the relation $\sigma =\omega \varepsilon_{0}\varepsilon_{r}^{\prime \prime }$. The Arrhenius plot for conductivity is also shown in Figure 6. The calculated activation energy $E_{a}$ is 0.87 eV, suggesting that conductive processes are associated and originated due to ionic conduction by segmental motions of polymer chains of PVB.

**Figure 7:**
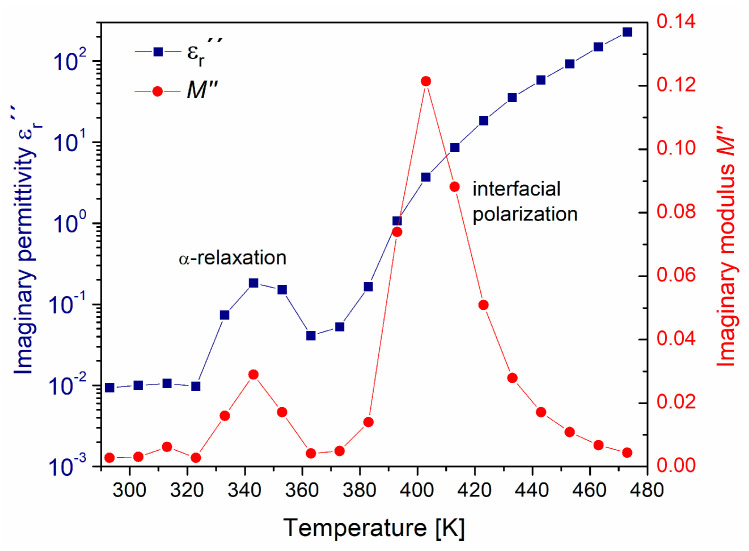
Comparison for $\varepsilon_{r}^{\prime \prime }$ and $M^{\prime \prime }$ at 100 Hz, as a function of temperature.

An isochronous graph was constructed at 100 Hz for $\varepsilon_{r}^{\prime \prime }$ and $M^{\prime \prime }$. The results obtained are presented in Figure 7. In this figure, in both isochronal curves at a temperature around 343 K, the peak associated with the α-relaxation is clearly identified. For a temperature around 393 K, only in the curve $M^{\prime \prime }$ is it possible to identify the relaxation peak associated with the phenomenon of interfacial polarization. This finding corroborates that by calculating the $M^{*}$, it is possible to differentiate the interfacial polarization from the α-relaxation and conductive processes. The analysis of $\varepsilon_{r}^{\prime \prime }$ and $M^{\prime \prime }$ presents identical information, but with different emphasis. The temperature at which the peaks associated with the interfacial polarization appear is an indicator of a transition from short-range to long-range mobility, corresponding to charge carriers trapped inside the PVB, thus requiring more energy to be released, in comparison with the cooperative motions of dipoles on the α-relaxation. At low temperatures, for the peaks associated with the α-relaxation, the electric charge carriers move short distances. At higher temperatures, such carriers can travel long distances, in the order of magnitude of the size of a repeating unit of PVB, which results in segmental movements. Moreover, the mobility of the charge carriers corresponding to the α-relaxation exhibits more localized mobility than the movements corresponding to the interfacial polarization.

### 3.4. Comparison between Experimental Data and Theoretical Predictions from the Fractional Debye Model

**Figure 8:**
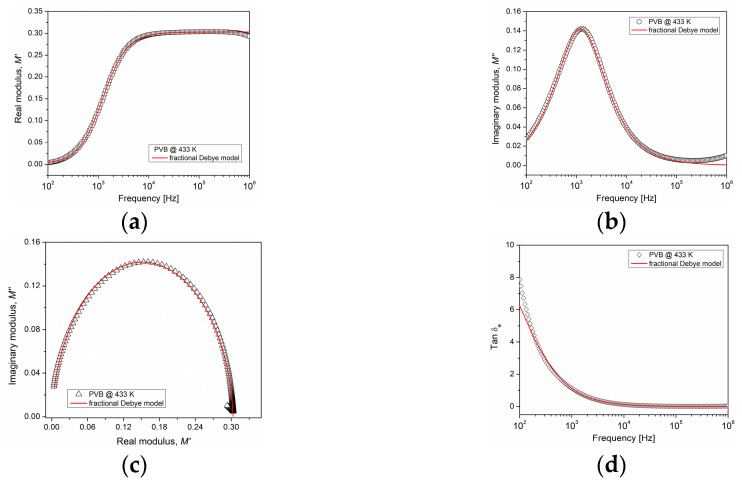
Comparison of the fractional Debye model with experimental data at 433 K. (**a**) real modulus, (**b**) imaginary modulus, (**c**) Cole-Cole plot, and (**d**) $\mathrm{Tan}\delta_{e}$ curve.

The experimental data of interfacial polarization in the frequency domain can be interpreted theoretically based on the fractional Debye model, Equations (7) and (8). The parameters that describe this model ($M_{s}$, $M_{\infty }$, $\tau_{M}$, γ) were obtained from the experimental curves for the PVB and are presented in Table 1. Figure 8 shows a good agreement between the theoretical predictions and experimental data obtained for $M^{\prime }$, $M^{\prime \prime }$, $\tan\delta_{e}$ and the Cole-Cole plot, at a temperature of 433 K. Only in the $\tan\delta_{e}$ plot did there occur a deviation observed at low frequencies, related to the contribution of the DC conductivity, which is not considered in the proposed model.

From Table 1, there is observed a strong dependence of the parameters on the temperature. Relaxation time $\tau_{M}$ decreases as the temperature increases, due to an increment in conductivity. Regarding the fractional order, this increases as the temperature increases, meaning that the PVB presents a conductive response. The fractional-order derivative is an indicator of the energy dissipated in the polymer. When the value of γ is close to 0, the mobility reflects a capacitor-like behavior, and when the value of γ is close to 1, the mobility indicates a resistor-like behavior. Therefore, interfacial polarization is strongly influenced by the conductive properties of PVB at temperatures above Tg.

### 3.5. Thermally Stimulated Discharge Current (TSDC)

**Figure 9:**
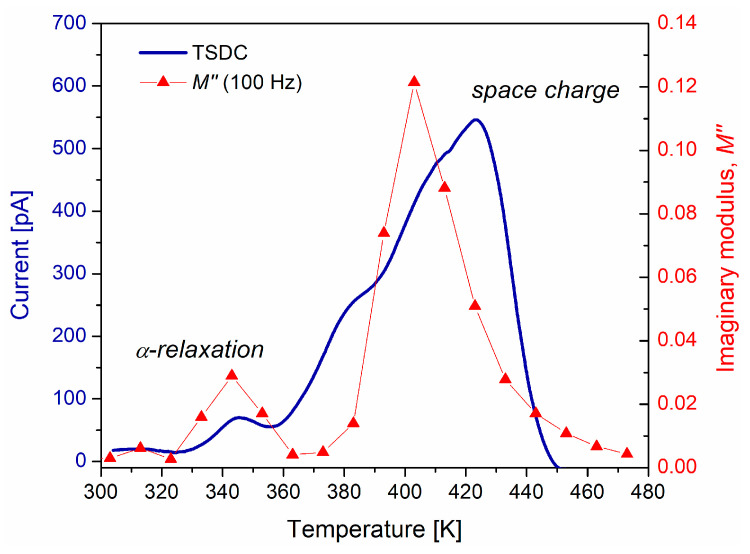
Comparison between TSDC and $M^{\prime \prime }$ (100 Hz) for the PVB.

Electrical current measurements were carried out using TSDC to corroborate that space charge or interfacial polarization manifests at temperatures above Tg. The current as a function of temperature was obtained and is presented in Figure 9. As a reference, in this figure the results of $M^{\prime \prime }$ are added. On the TSDC curve, two peaks were recognized at different temperatures. The first one, with the lowest amplitude (~70 pA), is located around 342 K and corresponds to the α-relaxation of the PVB. This corresponds to several electric dipoles aligned with the applied electric field. It can be stated that this peak is due to the stored electric charge from the elastic orientation of the electric dipoles. The second peak is observed at a 423 K, and its amplitude (~545 pA) is greater than that obtained for the α-relaxation. This peak can be associated with the space charge relaxation. When PVB is polarized above Tg, charge carriers are trapped. The thermal energy produces a release of trapped carriers from localized energy states. The trapping sites in amorphous polymers are favored by surface states, chain ends, and molecular disorder. Also, polar groups in polymer chains can act as traps. Consequently, this creates a scenario for the generation, transport, and diffusion of space charge. The differences between the results by $M^{\prime \prime }$ and TSDC are mainly due to the experimental nature of each analysis technique.

The area under the curve for each of the peaks is proportional to the electric charge that the PVB can store; it is evident that at high temperatures the storage capacity increases. Taking the ratio between the maxima of space charge and α-relaxation, a value of 7.78 is obtained. For $M^{\prime \prime }$ at 100 Hz, from Figure 5b, a value of 4.75 is obtained. This can be considered an indicator of the energy dissipated in these relaxation mechanisms. These results are important for potential applications of PVB such as thermo-electret, or sensor in the areas of electronic, mechatronic, and electrical engineering. To accomplish this, several authors suggest that PVB must have the form of a thin film, with an appropriate relative permittivity, low loss factor, facility of fabrication, high transparency, and good mechanical stability [43,44].

## 4. Conclusions

The properties that polyvinyl butyral PVB exhibits at temperatures around Tg are in concordance with the occurrence of interfacial polarization or space charge relaxation. At low frequencies and high temperatures, the conductivity of the polymer overlaps with the dielectric characteristics. We achieve the application of the complex electric modulus approach to separate the contribution of conductivity from relaxation phenomena. At temperatures higher than Tg, a relaxation phenomenon was obtained which is called space charge. The activation energy associated with this relaxation is 0.75 eV, which is influenced by the segmental movements of the PVB. In this study, it was proposed to use a fractional Debye model under the framework of the electric modulus. The theoretical predictions compared with the experimental results are good enough to demonstrate the capability of fractional derivatives for the interpretation of dielectric data under the electric modulus formalism.

## Figures and Tables

**Table 1:** Parameters obtained experimentally for the fractional Debye model.

	423 K	433 K	443 K	453 K	463 K	473 K
$M_{s}$	5 × 10^−4^	1 × 10^−4^	2 × 10^−4^	4 × 10^−4^	3 × 10^−4^	3 × 10^−4^
$M_{\infty }$	0.304	0.303	0.333	0.317	0.295	0.274
$\tau_{M}$ (s)	2.11 × 10^−4^	1.26 × 10^−4^	7.51 × 10^−5^	5.02 × 10^−5^	3.35 × 10^−5^	2.37 × 10^−5^
γ	0.932	0.958	0.985	0.989	0.992	0.994

## Data Availability

The data generated during the current study are available from the corresponding author upon reasonable request.
